# Design optimization of bidirectional arterial perfusion cannula

**DOI:** 10.1186/s13019-021-01500-3

**Published:** 2021-04-26

**Authors:** Saad Abdel-Sayed, Enrico Ferrari, Philippe Abdel-Sayed, Markus Wilhelm, Ludwig-Karl von Segesser, Denis Berdajs

**Affiliations:** 1 Department of Surgery and Anesthesiology, CHUV, Lausanne, Switzerland; 2 CardioCentro Ticino, Lugano, Switzerland; 3 Department of Musculoskeletal Medicine, CHUV, Lausanne, Switzerland; 4 Cardio-Vascular Surgery, Zurich University Hospital, Zurich, Switzerland; 5 Division of Cardiac Surgery, University Hospital, Basel, Switzerland

**Keywords:** Virtually wall-less cannula, Bidirectional cannula, Arterial cannulation, ECMO, ECLS, Leg ischemia

## Abstract

**Objectives:**

Determine if shortening the covered section of a self-expanding bidirectional arterial cannula, can enhance retrograde flow and thus reduce the risk of lower limb ischemia.

**Methods:**

Outlet pressure vs flow rate was determined for three cannulas types: a 15F self-expanding bidirectional cannula having a covered section of 90 mm, the same cannula but with a shorter covered section of 60 mm, and a Biomedicus cannula as control. The performances of all the cannulas were compared using a computerized flow-bench with calibrated sensors and a centrifugal pump. Water retrograde flow was determined using a tank timer technique. Anterograde and retrograde flow rate versus outlet pressure were determined at six different pump speed.

**Results:**

For each of the six pump speed, both bidirectional cannulas, 60-mm covered and 90-mm covered respectively, showed higher performance than Biomedicus cannula control, as demonstrated by higher flow rate and lower pressure. We also observed that for the bidirectional cannula with shorter covered section, i.e. 60 mm coverage, provides enhanced performance as compared to a 90-mm coverage. Finally, the flow rate and the corresponding pressure can be consistently measured by our experimental set-up with low variability.

**Conclusions:**

The new configuration of a shorter covered section in a bidirectional self-expanding cannula design, may present an opportunity to overcome lower leg ischemia during extra-corporal life support with long term peripheral cannulation.

## Introduction

Femoral arterial cannulation for arterial inflow is essential for cardiac surgical procedures, as well as for extra-corporal life support (ECLS) and extra-corporal membrane oxygenation (ECMO). The longer the femoral artery perfusion, the higher the risk of serious problems for distal perfusion. An eventual obstruction of the access vessel by traditional rectilinear cannulas design may cause irreversible hindrances such as amputation or even death. Several authors have stated an important risk of lower limb ischemic hitches after protracted cannulation of femoral artery, such as Hendrickson and Glower, who reported a rate of 11.5% after peripheral CPB [1]. Also, Foley et al. [2] and Huang et al. [3] have demonstrated that ECMO support is correlated with lower limb ischemic problem at rates as high as 26%.

Collateral blood flow is responsible for the viability of the lower limb. Incidence of lower limb ischemia is often related to deprived flow rate, leading to an obligation for fasciotomy or even amputation. A number of techniques have been proposed to prevent this potentially devastating complication, including the use of a downstream femoral perfusion catheter [1, 4–6] or an end-to-side femoral artery graft [7, 8]. However, these techniques are often difficult to perform, and in addition to the complexity of the procedure, they are not always consistent. Besides, bleeding complications and high risk of infection are often related to these techniques [9]. Thus, a new femoral cannulation system is necessary for clinical surgery and standard retrograde perfusion without reducing distal limb blood flow.

To overcome these difficulties, Smartcanula® LLC (Lausanne, Switzerland) developed a self-expandable bidirectional design for cannulation of femoral artery. This cannula has the enhanced performance of a nearly wall-less design as compared to the traditional rectilinear percutaneous cannulas. Besides, this cannula expands partially, i.e. at the insertion position only with regard to the vessel lumen, allowing thus parallel retrograde flow as the cannula body does not occupy the whole vascular lumen [10]. This novel bidirectional design allowed for important high flow rates at lesser driving pressures. Here, we present an optimized configuration of this bidirectional arterial cannula. The optimization development consists in shortening the narrow covered section of the cannula, which we assume could enhance peripheral perfusion, and thus diminish the risk of lower limb ischemia and subsequent complications during ECLS with peripheral cannulation [7]. Furthermore, we hypothesize that reducing size of the covered section may result in early cannulation and enhanced comfort without reducing the quality of peripheral perfusion.

## Material and methods

### Experimental setup

#### In vitro evaluation of forward flow rate and pressure

**Fig. 1:**
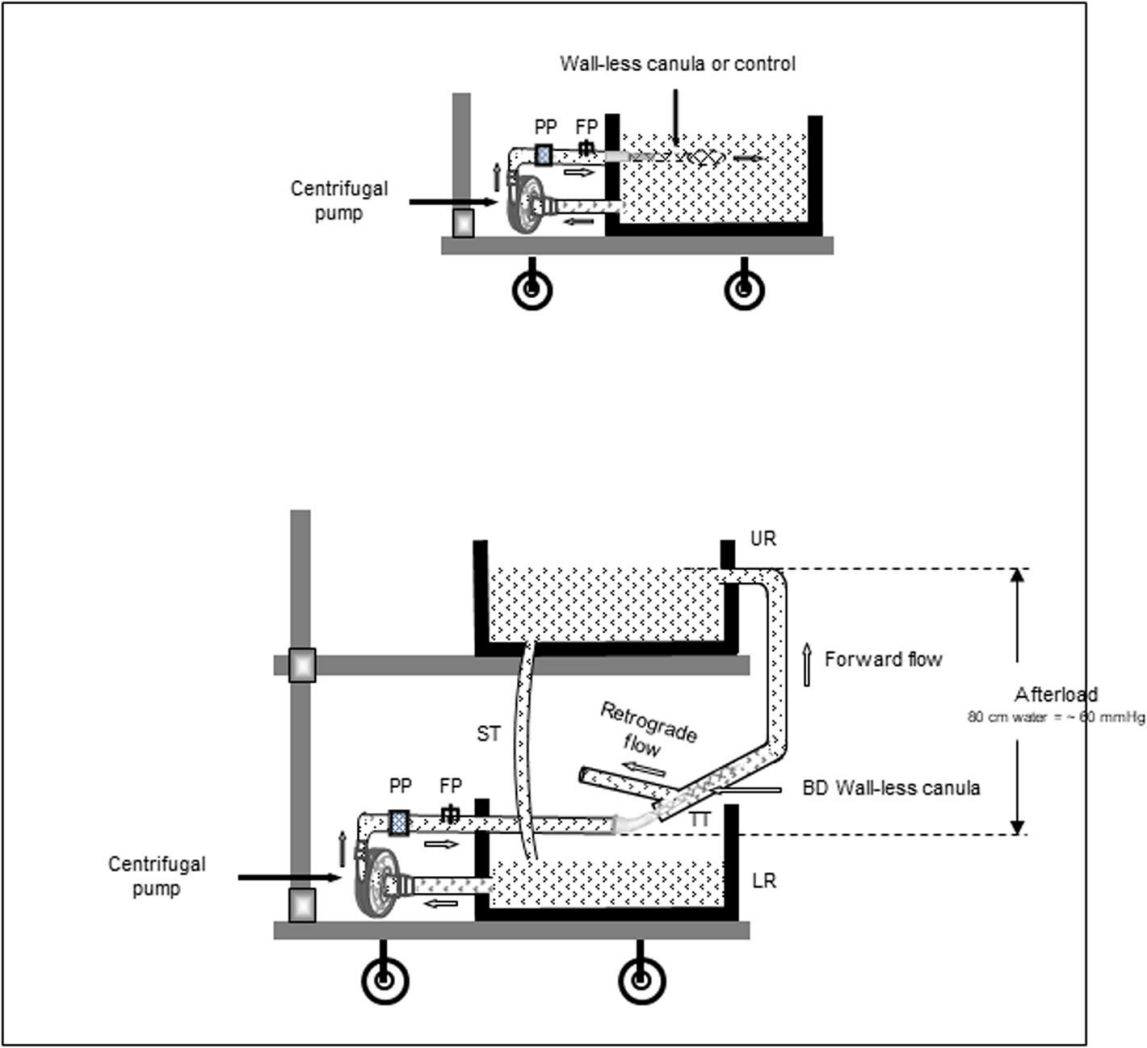
Diagram of the experimental set-up. Evaluation of cannula flow rate and driving pressure at different pump speed for bidirectional and control cannula (upper panel), and assessment of forward and retrograde flow distal to bidirectional cannula (lower panel). Upper reservoir (UR), lower reservoir (LR), silicon tubing (ST), test tubing (TT), pressure prob. (PP), flow prob. (FP)

Pressure values (P) and cannula anterograde flow rates (Q) were measured for both self-expanding cannulas and the rectilinear design control cannula using a simple in vitro circuit, as previously described [10]. Briefly, the in vitro circuit consisted of a hard-shell reservoir with the water being pumped to the tested cannula with a centrifugal pump through a 1 m long silicone” ½” tubing (Fig. 1, upper panel). Flow and outlet pressure were determined at six pump speed of 500, 1000, 1500, 2000, 2500, and 3000 RPM, using a bench calibrated flowmeter and adjusted pressure sensors, as well as a data acquisition system and LabView application.

#### Comparison of anterograde and retrograde flow distal to bidirectional cannula

The second in vitro circuit, allowing to measure both anterograde and retrograde flows, included two hard-shell reservoirs, an upper reservoir (UR) connected to a lower reservoir of the same size (LR) by a silicone tubing (ST) to keep the afterload volume of water constant. The vertical distance between the LR and the UR was set to 80 cm to correspond to a pressure of approx. 60 mmHg.

LR was connected to a centrifugal pump and silicon tubing as described for the first set-up. Here, the difference in the second circuit, remains in the fact that the tested cannulas were inserted into a test tubing (TT) of 18 F diameter and 20 cm long, containing an orifice on one side, 2 cm far from its end (at the site of cannula insertion) allowing to measure the retrograde flow, and connected to the UR at its other end (Fig. 1, lower panel). Water was pumped from the LR to the UR through the TT representing the anterograde flow. The water retrograde flow was determined from the orifice of the TT using the tank timer technique. Both, anterograde and retrograde flow versus outlet pressure were determined for the six RPM mentioned above. Furthermore, gap between the cannula and the wall of the simulated vessel was measured.

#### Cannulas

**Fig. 2:**
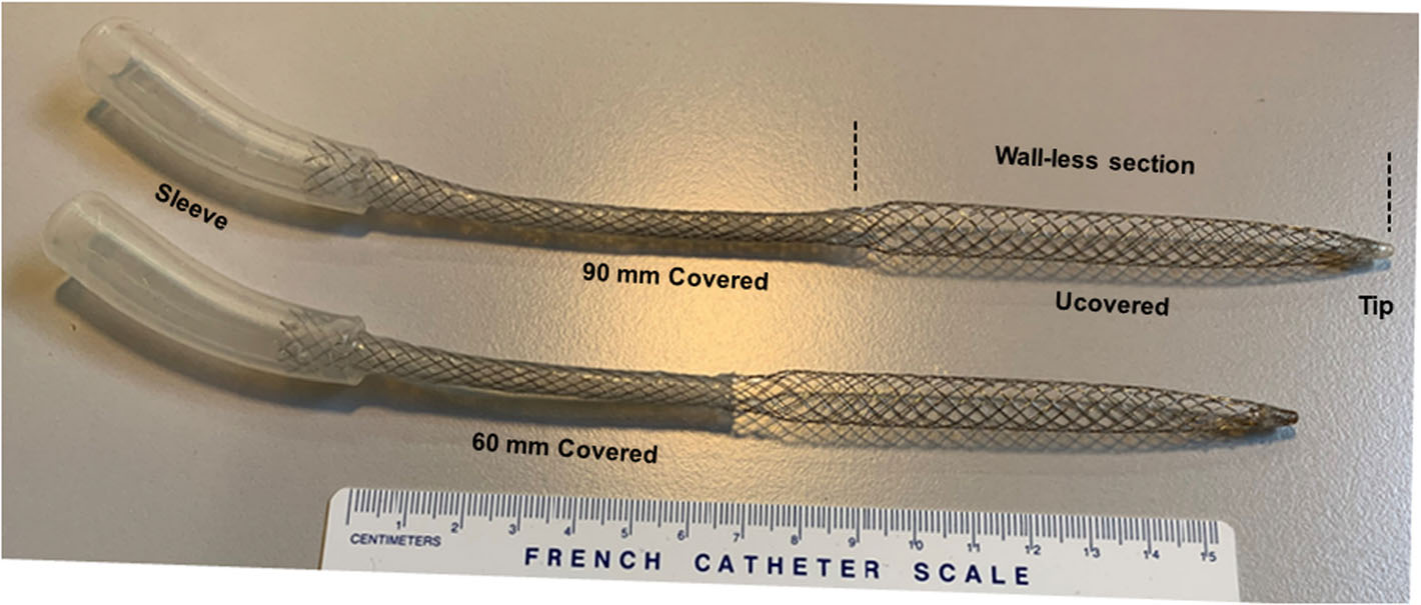
Smart bidirectional perfusion cannula 90 mm covered (upper cannula), 60 mm covered (lower cannula)

**Fig. 3:**
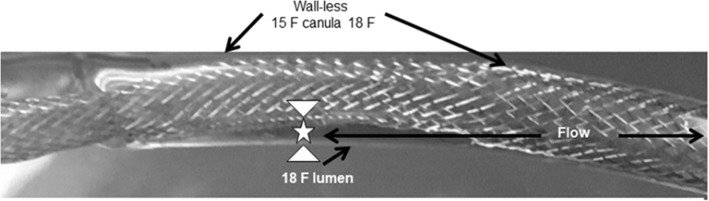
Bidirectional Smartcanula® inserted from the left to the right within a transparent tube mimicking femoral cannulation. The anterograde main flow intended for central perfusion is from the left to the right. A free space forms between the 15 F uncoated part of the cannula and the transparent tube. The fusiform uncovered section of the bidirectional cannula expands to the vessel wall (18 F) and provides retrograde flow to the periphery (limb)

Two smart bidirectional arterial cannula of 220 mm long were used in this study. As illustrated in Fig. 2, both cannulas have a meshed helical fusiform configuration, with respectively 90 mm and 60 mm long covered access section (15 F diameter for both), intended for non-occlusive cannula insertion, leading to a conical extra-corporeal *“3/8”* connecting sleeve. The uncovered fusiform section can be collapsed for a facilitated insertion and then be expanded to 24 F once inserted; in situ expansion allows free blood circulation in all directions (anterograde and retrograde), due to a free space between the bidirectional cannula and the artery wall, allowing for retrograde flow (Fig. 3). A third percutaneous Biomedicus arterial cannula (Medtronic, Tolochenaz, Switzerland) was used as a control for comparison.

#### Statistics

Each experimental measurement of flow rate and pressure was repeated six times for statistical analysis. Data were presented as means ± standard of deviation (SD). Precision was determined by the coefficient of variation (CV), which was calculated by the SD / mean. The unpaired Student t-test was used to compare the results of two cannulas. The standard two-way ANOVA test was used to compare between more than two cannulas. The significance level was *p* < 0.05.

## Results

### Comparison of anterograde flow rate versus pressure

**Fig. 4:**
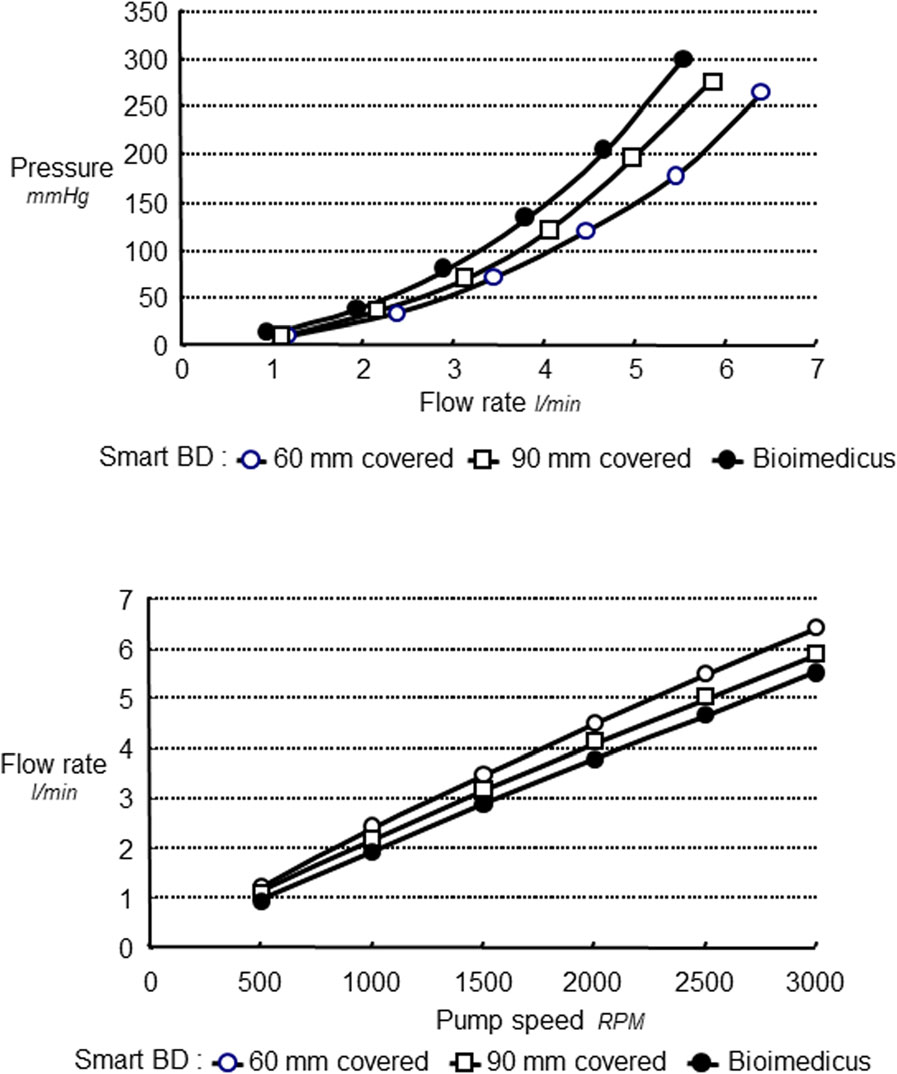
Comparison of forward flow rate versus pressure for bidirectional cannula 220 mm long, 60 mm and 90 mm covered section and Biomedicus cannula (upper panel), and forward flow rate versus pump speed (lower panel)

Self-expanding arterial cannulas, 60 mm and 90 mm covered access section, clearly demonstrated higher forward Q and lower *P* values with regard to the control cannula (Fig. 4, upper panel). The forward flow rate steadily increased with the increase of pump speed (Fig. 4, lower panel). Improved anterograde flow rate to each revolution per minute for both self-expanding bidirectional cannulas 60, was on average 15 and 9% respectively compared to the standard control cannula (Table 1).

**Table 1:** Comparison of flow rate and positive pressure at different pump speed

Cannula	RPM	500	1000	1500	2000	2500	3000
Biomedicus	Pressure *mmHg*	10.27 ± 1.01	36.11 ± 3,29	76.45 ± 3,16	130.00 ± 5,95	201,30 ± 15,12	298,33 ± 26,43
^a^BD 90 mm ^b^BD 60 mm		8,86 ± 0,15 8,35 ± 0,40	30,44 ± 0,39 32.39 ± 0.30	68.25 ± 14.86 68,62 ± 0,88	120,01 ± 0,50 119,25 ± 0,11	194,93 ± 0,33 180,00 ± 18,34	275,90 ± 0,47 261,83 ± 0,64
Biomedicus	Flow rate	0.94 ± 0.01	1.93 ± 0,01	2,88 ± 0,01	3,77 ± 0,01	4.65 ± 0,01	5.52 ± 0.01
^a^BD 90 mm ^b^BD 60 mm		1,10 ± 0,01 1.17 ± 0,01	2,15 ± 0,01 2,37 ± 0.01	3,13 ± 0,01 3,44 ± 0,01	4.05 ± 0.01 4,46 ± 0,01	4,96 ± 0,01 5.44 ± 0,01	5.85 ± 0,01 6,38 ± 0,01

Mean ± SD; *n* = 6

***p < 0.0001 for Bidirectional cannula versus Control cannula

^a^Bidirectional 90 mm covered

^b^Bidirectional 60 mm covered

### Comparison of retrograde flow rate versus pressure

**Fig. 5:**
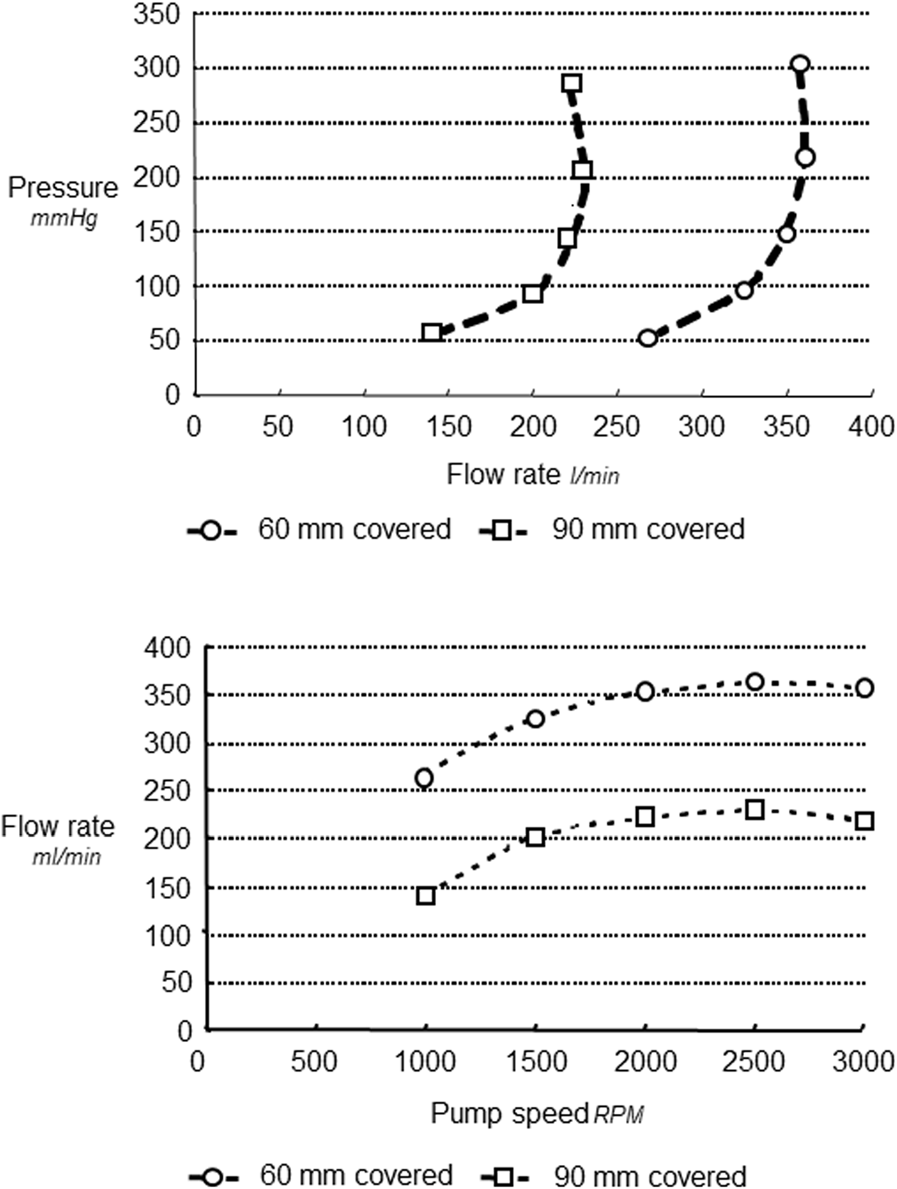
Comparison of retrograde flow rate versus pressure for bidirectional wall-less cannula 220 mm long, 60 mm and 90 mm covered (upper panel), and retrograde flow rate versus pump speed (lower panel)

**Fig. 6:**
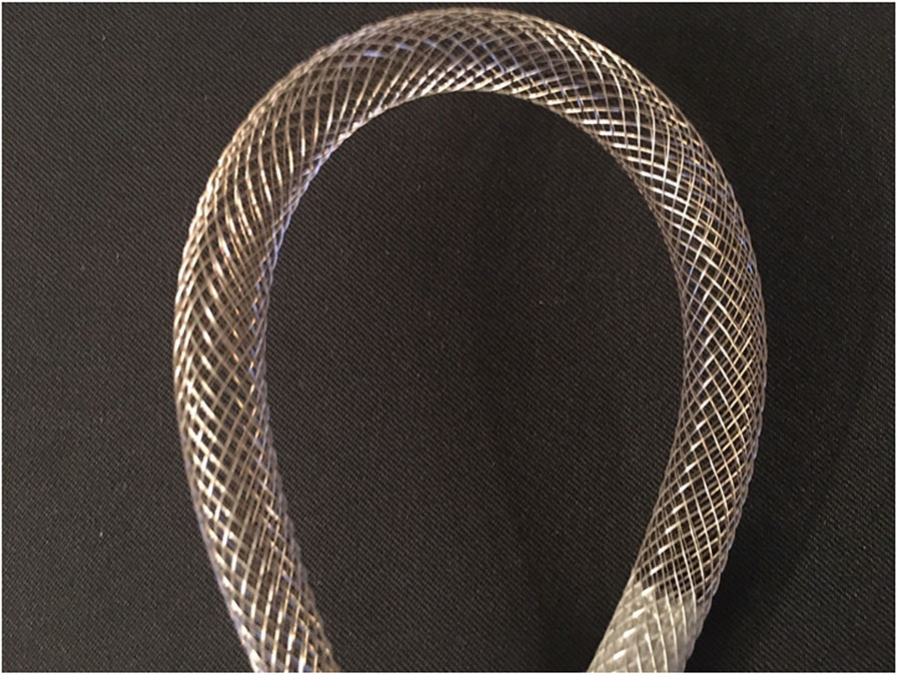
Curved Smartcanula demonstrating its kink resistance. The latter is due to its double helix design

Results clearly show that the 60 mm covered access section bidirectional cannula provided higher retrograde flow rate as compared to the 90 mm covered. At 100 mmHg driving pressure, the corresponding retrograde flow rate values were 325 ± 0.2 and 200 ± 2 m l/min for the 60 mm and 90 mm covered bidirectional cannulas, respectively (Fig. 5, upper panel). Retrograde flow rate steadily increased with the rise of pump speed. At 2000 RPM, retrograde flow rate was 309.50 ± 46 and 216.75 ± 17 ml/min for the 60 mm and 90 mm covered respectively (Fig. 5, lower panel). Regular design arterial Biomedicus control with the performance closest to the test cannula did not show retrograde water flow. The residual lumen allowing for retrograde flow accounted for 9.81 ± 0.83 mm^2^ for bidirectional versus non-measurable for control. The kinking assessment of self-expanding cannulas tests confirmed for all self-expanding cannula diameters (12F–36F), that the double helix design is extremely kink resistant (180^o^ and more) as shown in Fig. 6.

## Discussion

Overall, both self-expanding bidirectional showed better forward flow rate to each revolution per minute when compared to the standard control cannula. This superior forward flow rate with the virtually wall-less cannulas is probably due to their tinny wall thickness (0.36 mm) as compared to the wall leanness of percutaneous cannula (> 0.60 mm). This difference in wall thickness affects the effective cross-sectional area. The superiority of the bidirectional cannula was revealed with less positive pressure for each forward flow rate, compared with the regular control cannula (Fig. 4, upper panel) (*p* < 0.001). In vivo perfusion with high positive pressure in rectilinear cannula design increases the risk of hemolysis and embolism formation.

Furthermore, the short bidirectional cannula design (60 mm of the 15 F covered access section), showed significantly higher forward flow rates at lower driving pressures as compared to the 90 mm covered. For a driving pressure of 100 mmHg, typical of a clinical situation, the corresponding flow rate is around 4.1 l/min for bidirectional 60 mm covered and versus 3.8 l/min for bidirectional cannula 90 mm covered, versus 3.2 l/min for control cannula corresponding to around 128% for 60 mm versus control and 108% for 60 mm versus 90 mm**.** The advantages shown for the bidirectional 60 mm covered can be repeated at all pump speeds with minimal variations (CV between 1 and 4% for six repeated measures). Thus, shortening the 15 F free section length of the bidirectional cannula permits for higher flow rates at lower driving pressures. For a flow rate of 4.5 l/min the corresponding driving pressure is 180 mmHg for the regular design control of the same diameter versus 150 mmHg for bidirectional cannula 90 mm, versus 119 mmHg for bidirectional 60 mm covered, equal to around 150% for control versus 60 mm covered, and 126% for 90 mm covered versus 60 mm covered. (Fig. 4, upper panel). In addition, bidirectional 60 mm covered showed 160% more retrograde flow rate as compared to the 90 mm covered. With our experimental system, the rectilinear design Biomedicus control did not show retrograde flow rate.

In clinical practice, a considerable bigger size of a traditional rectilinear percutaneous cannula would be needed to reach the same flow rate at the same driving pressure, due to less or no space for retrograde flow to the limb. Our results indicate that a considerable smaller cannula diameter of bidirectional design with 15 F covered access section can be used to attain a given target flow rate and a physiologic driving pressure.

In the clinical setting, bidirectional cannula design is based on the “collapsed insertion and *in situ* expansion principle”, which has been confirmed to be reliable [8]. Thus, for the clinical application no concerns about the insertion and the removal of a bidirectional cannula with its fusiform segment larger than the cannula diameter at the site of insertion [10] (Fig. 2). This function is achieved by extending the fusiform cannula section with a mandrel [11]. Similarly, the bidirectional canula can be removed easily, because it collapses with simple traction. Digital compression for caudal bleeding.

The results of this study were performed with water as the testing medium, which might be considered as a limitation when compared to tests performed with blood, for describing results relevant to the clinical setting. Thus, we questioned whether the outperformance of the cannula would be identical using blood instead of water, and if the higher viscosity of the blood would change its performance. Our preceding studies demonstrated, that the viscosity of the blood decreased flow by about 10 and 6% less for rectilinear percutaneous cannulas and virtually wall-less cannulas respectively [12], similar to the bidirectional design presented here. Also, Broman el. al [13, 14] showed that when blood with hematocrit of 27% was used, the venous drainage pressure was steadily higher for a given flow than when water was used. Broman and co-authors demonstrated that blood flows with single-lumen return arterial cannulas for peripheral ECMO tested were lower than those provided by manufacturers using water. We think that water is a good testing medium for the cannula performance. The main advantage of water as test medium for cannula performance assessment is its reproducibility. This also explains why water is usually used as the industry standard medium for comparative to compare cannula performance validation. For the present set-up, the water transparency was necessary for assessment of the residual lumen resulting in retrograde flow.

Severe atheromatic disease access vessel or kinked access vessels may restrict the self-expanding mechanism of the bidirectional cannula and therefore the flow may also be delayed in one or both directions. The kinking assessment of self-expanding cannulas tests confirmed for all self-expanding cannula diameters (12F–36F), that the double helix design is extremely kink resistant (180^o^ and more) as shown in Fig. 6. However, a traditional rectilinear cannula with a fixed outer diameter may not even be inserted. The use of per-procedural ultrasound is a good tool in such complex situations as described above. The latter is helpful for target vessel identification and also guide-wire position clarification. For the bidirectional cannula, ultrasound allows in addition for identification of the flow direction by the means of the Duplex Color Doppler function [8]. We conclude bidirectional flow for ECLS and ECMO can be achieved on the arterial side with cannulas having a 15 F section, that does not occupy completely the access vessel for a retrograde flow, and which performance can be optimized by shortening its covered section. We consider that this type of cannula can also be used and optimized for MICS [15–17]. In vivo studies are planned as the next step for confirmation of these findings prior to clinical application.

## Conclusion

The enhanced performance in a bidirectional self-expanding cannula with in a shorter covered section, may present an opportunity to overcome lower leg ischemia during ECLS with long term peripheral cannulation.

## Data Availability

All data generated or analyzed during this study are included in this published article and its supplementary information files.

## Abbreviations

F: French (1 F = 3 mm)
ECMO: Extracorporeal membrane oxygenation
ECLS: Extracorporeal life support
P: Pressure
Q: Flow rate
RPM: Round per minute
UR: Upper reservoir
LR: Lower reservoir
ST: Silicone tubing
TT: Est. tubing
BD: Bidirectional
SD: Standard of deviation
CV: Coefficient of variation
